# Avoiding exercise mediates the effects of internalized and experienced weight stigma on physical activity in the years following bariatric surgery

**DOI:** 10.1186/s40608-018-0195-3

**Published:** 2018-07-02

**Authors:** SeungYong Han, Gina Agostini, Alexandra A. Brewis, Amber Wutich

**Affiliations:** 1 Mayo Clinic/Arizona State University Obesity Solutions, 1000 Cady Mall Suite 164, Tempe, AZ 85287 USA; 2 School of Human Evolution and Social Change, Arizona State University, 900 Cady Mall, Tempe, AZ 85287 USA

**Keywords:** Obesity, Bariatric surgery, Stigma, Physical activity, Exercise

## Abstract

**Background:**

People living with severe obesity report high levels of weight-related stigma. Theoretically, this stigma undermines weight loss efforts. The objective of this study is to test one proposed mechanism to explain why weight loss is so difficult once an individual becomes obese: that weight-related stigma inhibits physical activity via demotivation to exercise.

**Methods:**

The study focused on individuals who had bariatric surgery within the past 5 years (*N* = 298) and who report a post-surgical body mass index (BMI) ranging from 16 to 70. Exercise avoidance motivation (EAM) and physical activity (PA) were modeled as latent variables using structural equation modeling. Two measures of weight stigma, the Stigmatizing Situations Inventory (SSI) and the Weight Bias Internalization Scale (WBIS) were modified for people with a long history of extreme obesity for use as observed predictors.

**Results:**

Exercise avoidance motivation (EAM) significantly mediated the association between both experienced (SSI) and internalized (WBIS) weight stigma and physical activity (PA) in this population.

**Conclusion:**

Exercise avoidance motivation, influenced by weight stigma, may be a significant factor explaining the positive relationship between higher body weights with lower levels of physical activity.

**Electronic supplementary material:**

The online version of this article (10.1186/s40608-018-0195-3) contains supplementary material, which is available to authorized users.

## Background

Weight-related stigma is frequently reported by people living with the highest levels of obesity, and its generally negative effects on health and wellbeing are well documented [1–9]. Larger-bodied individuals more frequently endure both direct and indirect forms of felt (experienced) weight stigma in many common environments, including work, school, medical facilities, and government centers [10]. Importantly, people with severe obesity (BMI ≥ 35) are more likely than those of normal weight to internalize the weight-related stigma expressed by others (i.e., to self-stigmatize) over their life course [10, 11]. Internalized weight stigma (sometimes termed self-stigma or self-directed stigma), is the extent to which a person reports feeling of low social value due to his or her current or former weight status. Internalized stigma arises because an individual agrees with the publicly held, usually negative, stereotypes used to characterize a group or population (e.g., overweight individuals) and adopts these negative views about the self or of his/her capabilities [12]. Internalized stigma more strongly affects both self-esteem and self-efficacy than do other forms of stigma (e.g., public stigma) across many different contexts, and, further, has long-lasting effects [13]. There is growing evidence that such stigma predicts difficulty in both reaching and maintaining a healthy weight, especially for individuals with a higher BMI. For example, higher levels of internalized weight stigma are associated with a number of behavioral changes, including greater calorie consumption, demotivation to diet, and binge eating disorder, all factors that can stymie weight loss [4, 14–16]. Furthermore, individuals who report high levels of internalized weight stigma are less likely to show improvement when they do seek treatment for disordered eating [17].

Weight stigma also impacts behaviors and attitudes toward physical activity. This can manifest as a reluctance to engage in specific, public forms of activity (e.g., gyms, swimming pools, or social sports teams) or as exercise avoidance more generally [1, 10, 18, 19]. For example, Vartanian and Shaprow [20] found a significant effect of experienced weight stigma on exercise avoidance in female college students which predicted less frequent bouts of strenuous and moderate physical activity. These effects were exacerbated among those already overweight and obese, or who believed themselves to be so. In a study of adult females with a body mass index (BMI) at or over 25, Pearl, Puhl, and Dovidio [21] found a significant effect of experienced weight stigma on exercise behavior and, further, that internalized weight stigma partially mediated this association. Similarly, among males who perceived themselves as overweight or obese, internalized societal attitudes toward appearance (including anti-fat attitudes) moderated the association between experienced weight stigma and exercise avoidance, which itself was negatively associated with self-reported strenuous exercise [18]. Among health program enrollees, Mensinger et al. [22] discovered that individuals who initially reported lower internalized weight stigma also had greater enjoyment of and engagement in moderate intensity physical activity (PA) while those individuals with high levels of internalized stigma showed little change to either over 6 months. While exercise avoidance was not a specific target of this study, these more negative attitudes towards exercise may express as a general reluctance to engage in activity (i.e., exercise avoidance). In total, these studies suggest that the mechanisms fueling exercise avoidance include direct and immediate concerns of judgment or mistreatment, but also that internalized stigma may undermine self-efficacy [17, 21–23] to create a “why try” mentality when it comes to weight loss, particularly when an individual is overweight [10, 19, 21, 24–27].

To expand the evidential basis of how stigma shapes exercise avoidance, here we focus on a population of bariatric patients in the years after their surgery. This is a population where the connections between stigma, obesity, and exercise avoidance decisions should be especially apparent. Bariatric (weight loss) patients decide to have surgery after difficult and long, often lifetime, struggles with both high body weight and the stigma it engenders [28]. To qualify for surgery, patients must often have a BMI of 40 or above, or a BMI of 35 or more (technically severe obesity) with associated co-morbidities. Studies show that individuals who elect to have surgery commonly report long-term exposure to stigmatized treatment by others, such as being stared at, enduring negative statements from children or other adults, and being overlooked for certain opportunities (e.g., work promotion) [1, 28, 29]. Evidence suggests that social stigmas are more severe and more likely to manifest as internalized stigma or self-blame when an individual is deemed personally responsible for his/her stigmatizing condition (i.e., being overweight), the effects of which are long lasting and may persist even if the stigmatizing variables change. [15, 30, 31].

After surgery, clinicians set guidelines for physical activity to assist with both weight-loss and maintenance [32–34]. However, Bond et al. [35] found no significant difference in moderate-to-vigorous physical activity (MVPA) tracked via accelerometer between patients assessed at presurgical and postsurgical (6 month) windows. For individuals like bariatric surgery patients who have lost a substantial amount of weight, a high level of physical activity is crucial to maintain their reduced weight status and prevent regain [36–39]. And yet bariatric surgery patients are less likely to comply with physical activity guidelines than other guidelines, including dietary ones, with rates of exercise program noncompliance increasing after surgery [40–42]. A lack of motivation and related psychological factors have been proposed to explain these persistently low levels of physical activity [43]. Given this, connecting stigma to exercise avoidance in the years after surgery has important practical applications for understanding (and preventing) weight regain in bariatric patients, particularly given the greater success in long-term weight loss for patients who engage in even low levels of regular physical activity [33, 44].

## Methods

All participants in this study were identified on the basis of age (greater than 21) and having had bariatric surgery within the previous 5 years in a single large hospital system. Final sample composition was consistent with the hospital’s bariatric surgery population generally [45]: patients were primarily female (77%) and Non-Hispanic White (93%). The majority of patients reported undergoing roux-en-y gastric bypass surgery (74%), followed by a sleeve gastrectomy (17%), duodenal switch with biliopancreatic diversion (5%), and laparoscopic gastric binding (2%). Two percent of respondents were uncertain which surgical procedure they received. Patients ranged in age from 19 to 82 years and had a BMI greater than 35 at the time of surgery (see Table 1). Data were generated using paper surveys disseminated via mail in 2015 with follow-up phone calls to encourage participation and assist with completion as needed. The survey was paired with a long-term ethnographic study of bariatric patients’ experiences with stigma [2, 28]. These associated qualitative interviews informed the survey tool development. All procedures were approved by the relevant university and hospital human subjects review boards. For a mail–disseminated survey of this type, the response rate of 30% (298 out of 994 patients) was excellent.

**Table 1:** Summary Statistics of Outcome, Mediator, Predictors, and Covariates

	N	N miss	Mean	S.D.	Min	Max
*Physical activity (PA)*						
Non-vigorous physical activity, days	285	13	4.65	2.13	0	7
Non-moderate physical activity, days	283	15	3.84	2.26	0	7
No walk > 10 mins, days	283	15	2.17	2.35	0	7
*Exercise Avoidance Motivation (EAM)*						
Uncomfortable going to a gym	287	11	2.20	1.68	1	7
Too many thin people at a gym	286	12	1.94	1.60	1	7
Embarrassed to use gym equipment	282	16	2.04	1.60	1	7
Embarrassed to exercise in public places	293	5	1.70	1.34	1	7
Experienced weight stigma (SSI)	219	79	4.27	7.10	0	66
Internalized weight stigma (WBIS)	283	15	2.33	0.45	1.2	3.5
*Covariates*						
Current BMI	291	7	30.60	6.53	16.0	70.4
Weight loss (%)	293	5	−33.36	9.96	−67.9	−1.7
Time since surgery (months)	293	5	20.92	12.44	0	60
Male	289	9	0.23	0.42	0	1
Age	288	10	53.76	12.72	19	82
University or above	295	3	0.45	0.50	0	1
Household income > $10,000	289	9	0.29	0.45	0	1

The total sample size is 298

**Fig. 1:**
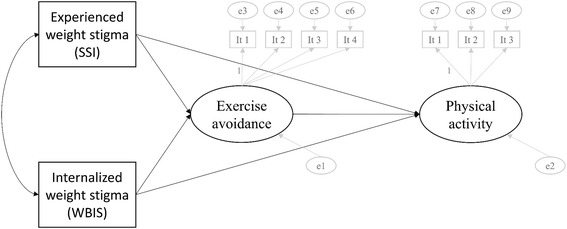
Diagram of Structural Equation Model for Analysis. Note: “It” stands for item; identification (*p* = 120 and q = 62, d.f. = p-q = 120–62 = 58)

For data analysis, a structural equation model (SEM) in Mplus 7.4 was used. The visual model of the analysis is presented in Fig. 1, with ovals signifying the latent variables of exercise avoidance motivation (EAM) and physical activity (PA), and boxes reflecting observed variables (4 constructs of exercise avoidance, 3 constructs of physical activity, as well as experienced (SSI: Stigmatizing Situations Inventory) and internalized (WBIS: Weight Bias Internalization Scale) weight stigma). This analytical approach was suitable for our data and superior to other such methods (e.g., a series of multiple logistic or ordinary least square regressions) in that (1) SEM incorporates random and systematic measurement error in the indicators of EAM and PA; (2) the indirect effects of either experienced or internalized weight stigma through exercise avoidance could be tested, each controlling for the other; and (3) a full sample (*N* = 298) could be used via the full information maximum likelihood (FIML) method to handle missing data in Mplus 7.4 [46]. In addition, to deal with the imbalance of confidence limits due to the non-normal distribution of the indirect effect, bias-corrected bootstrap confidence intervals after 5000 replications were used to interpret the results. The outcome, one mediator, two predictors, and seven covariates used for the SEM were measured as follows.

### Physical activity as an outcome

We used a structural equation framework wherein three items of physical activity were used to construct a latent variable measuring overall physical activity: (1) the number of days of no vigorous physical activity per week, (2) the number of days of no moderate physical activity per week, and (3) the number of days not walking more than 10 min per week. Higher values, therefore, indicated lower physical activity. The associations between the latent variable and those three items are visualized in Fig. 1. This physical activity index has a medium level of internal consistency using all three items (Cronbach’s alpha = .68).

### Exercise avoidance as a mediator

The survey adopted eight questions from Vartanian and Shaprow [20] that were used to construct an exercise-avoidance motivation (EAM) scale. Each question was scored from zero (“not at all true”) to seven (“completely true”), and patients reported on their experiences within the prior 3 months. The EAM scale has been employed previously with obese and general populations [18, 20], but has not been validated for bariatric populations specifically. In addition, despite the high level of internal consistency for the index using all eight items (Cronbach’s alpha = .86), a maximum of four items are recommended to construct a latent variable [47]. Accordingly, four items were selected based on the results of a common factor analysis allowing two factors (average eigenvalue used as the criterion) to be correlated with each other (oblique PROMAX rotation, results provided upon request). The four questions asked whether a respondent felt (1) uncomfortable going to a gym, (2) that there were too many thin people at the gym, (3) embarrassed to use gym equipment, and (4) embarrassed to exercise in public places, including gyms. Contextually, all four questions refer to gyms, so theoretical internal consistency is high. The associations between the latent variable and these four items are also visualized in Fig. 1. Higher values for the latent variable, therefore, indicated a greater tendency to avoid exercise.

### External and internal weight stigma measures as predictors

Two indices were used to assess a patient’s level of experienced and internalized weight stigma: one focused on experiences of being stigmatized by others (SSI) while the other captured respondents’ weight-related self-judgment (WBIS). The Stigmatizing Situations Inventory (SSI) measured experiences of weight-related stigma, sources of stigma (e.g., from family or strangers), and encounters with weight-related physical barriers (e.g., ill-fitting seatbelts) reported by participants. It captured how often people recognize various encounters with stigma in their everyday lives. The SSI index has been applied to both those populations seeking bariatric surgery [18] and those seeking non-surgical weight loss interventions [7]. We reduced the SSI items from 50 [1] to 29 to limit respondent burden. Each item was scored from zero (“never”) to three (“several times”), and patients reported on experiences during the 3 months prior to the survey. All items were categorized into 11 stigmatizing situations, the sum of which was used to construct the SSI index. It should be noted that for one stigmatizing situation, “being physically attacked,” all respondents answered the same: that they had never experienced this situation before. The SSI index was only valid if there are no missing items. The valid range of our constructed index was between 0 and 90, and the level of internal consistency (excluding “being physically attacked” due to no variance) was high (Cronbach’s alpha = .82).

To assess internalized weight stigma experienced in the 3 months prior to survey, 13 questions were chosen from the original 19 questions used by Durso and Latner [48] to construct their 11-item Weight Bias Internalized Scale (WBIS). The phrasing of these questions were slightly modified so they would be more applicable to our participants, each of whom had a long history of extreme obesity. To test whether all items reflected the same construct, SAS 9.2 was used to conduct a principal component analysis with varimax rotation on the 13 items following Durso and Latner [48]. Results confirmed that these items clustered around two components (eigenvalues > 1), with component 1 explaining most of the variance (73% and eigenvalue = 3.84). Due to the low variance explained by the second component (eigenvalue = 1.58) and the single dimensionality of the hypothesized construct, Mplus 9.2 was used to conduct confirmatory factor analysis to assess whether the two components could be merged. Based on these results, six items were dropped from the constructed WBIS because of non-significant or low factor loadings (< 0.35). Therefore, an abbreviated 7-item WBIS was used to reflect internalized weight stigma for subsequent analyses (see the Additional file 1 for the description of the final questions selected) [49]. The final 7-item WBIS was only created for cases where all seven items were complete. The level of internal consistency was high (Cronbach’s alpha = .87).

### Covariates

Multiple covariates potentially associated with EAM and PA were controlled for. In addition to body mass index (BMI) at the time of the survey, percent weight change between the time of surgery and the time of survey completion was also controlled in the model. It should be noted that because this survey was part of a larger social science study of patient’s lived experience and because medical records were not accessible, direct measurement of weight was not possible. Other covariates included age, gender, time since surgery (months), education (university educated or above), and household income (over $100,000 or not). Due to low variation within the sample, we did not include ethnicity as a covariate.

## Results

### Summary statistics

Moderate physical activity (PA) was more common than vigorous physical activity among patients after bariatric surgery, and patients walked at least 10 min per week for 2 days on average (see Table 1). All four items of the latent variable of exercise avoidance motivation (EAM) showed relatively low values with wide variance. Bariatric surgery patients had not experienced many stigmatizing situations (SSI) during their lives and showed a relatively low level of internalized weight stigma (WBIS). Results for covariates showed, on average, that the bariatric patients in the sample were middle aged (54 years old), primarily female, highly educated and affluent, that they lost 33% of their weight at the time of surgery, that they still had a high body mass index (BMI > 30), and that surgery was performed more than 21 months before the survey. The percent weight change was not significantly correlated with the three items measuring physical activity. However, it was significantly correlated with whether a respondent felt that there were too many thin people at the gym (coef. = 0.19, *p*-value = 0.0015) and whether they were embarrassed to use gym equipment (coef. = 0.13, *p*-value = 0.0315).

### Structural equation model results

**Fig. 2:**
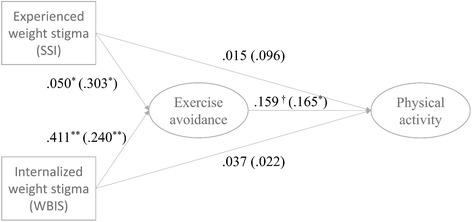
Direct Effects of SSI and WBIS on Exercise Avoidance and Physical Activity, Structural Equation Model Analysis Results. Note: Standardized direct effects in parentheses;: * *p* < 0.05; ** *p* < 0.01; *** *p* < 0.001, two-tailed; Covariates, including current BMI, time-since-surgery, gender, age, education, and household income, are controlled for in the model, but omitted

The main results of the structural equation model are summarized in (Fig. 2, see the Additional file 2 for the full results). One of our primary interests was whether the effect of weight stigma on physical activity could be mediated by exercise avoidance. All covariates (exogenous variables omitted in Figs. 1 and 2) were allowed to correlate, and they were uncorrelated with all the errors or disturbances. The test statistic from the Chi-square test comparing the suggested model with the saturated model was 106.522 with 58 degrees of freedom (*p*-value = .0001), but this was likely due to the relatively large sample size (*N* = 298). The other fit statistics indicated good fit to the data with RMSEA close to 0.05 (=0.053 with 90% C.I. [0.037; 0.069]) and CFI and TLI larger than .90 (CFI = 0.933 and TLI = 0.904).

The unstandardized and standardized estimated direct effects are summarized in Fig. 2. Both experienced (SSI) and internalized (WBIS) weight stigma had significant and positive effects on exercise avoidance motivation (EAM) at the *p*-value .05 level. On the other hand, neither SSI nor WBIS showed a significant *direct* effect on physical activity (PA). Only EAM showed a marginally significant effect on PA (*p*-value = 0.063). The absence of a direct effect of experienced and internalized weight stigma on PA does not necessarily mean that indirect effects do not exist. The estimated direct, indirect, and total effects of each type of weight stigma after 4856 successful bootstraps (5000 attempts) are summarized in Table 2. The numbers are unstandardized estimates. The bias-corrected 95% confidence intervals confirmed that EAM significantly mediated the associations of PA with both SSI and WBIS. In detail, there was a 0.066 unit increase in the latent variable of PA through EAM for every unit increase in WBIS. The scale of a latent variable is the same as the scale of the indicator with a factor loading fixed at one, so the scale of the latent variable measuring PA in our model was days per week. In other words, one unit increase in WBIS was, on average, associated with 0.066 days per week (bias-corrected 95% C.I. between 0.013 and 0.190) increase in physical activity. This indirect effect explained 64.7% of the total effect (=0.066/0.102*100). While the magnitude of the effect size may not seem overtly substantive, comparing the lowest and the highest levels of observed WBIS in the data (ranging from 1.2 and 3.5, see Table 1) indicated a clear difference in physical activity of 0.152 days per week. EAM also mediated the association between experienced weight stigma (SSI) and PA. In detail, one unit increase in SSI was, on average, associated with 0.008 days per week (bias-corrected 95% C.I. between 0.001 and 0.030) increase in physical activity. This indirect effect explained 35% of the total effect (=0.008/0.023*100). And the standardized coefficients of two indirect effects indicate that two weight stigma indices have similar impacts on PA through EAM (see Fig. 2).

**Table 2:** Indirect, Direct, and Total Effects of Weight Stigma on Physical Activity, Bootstrapping Results

	Coefficient	S.E.	Bias-corrected 99% C.I.
Experienced weight stigma (SSI)			
Indirect effect	**0.008**	0.007	[0.001; 0.030]
Direct effect	0.015	0.016	[−0.011; 0.053]
Total effect	0.023	0.015	[0.000; 0.058]
Internalized weight stigma (WBIS)			
Indirect effect	**0.066**	0.039	[0.013; 0.190]
Direct effect	0.037	0.126	[−0.185; 0.325]
Total effect	0.102	0.122	[−0.091; 0.393]

N = 298 and the number of completed replications = 4856 (out of 5000 requested); **bold** if the result is significant at the *p*-value 0.05 level; *CI* stands for confidence intervals, *SE* stands for standard errors; covariates, such as current BMI, weight loss, time since surgery, gender, age, education, and household income, are controlled for in the model, but omitted; the developers of Mplus recommend reporting non-significance when either the lower bound or the upper bound of the C.I. is reported as 0.000

## Discussion

In total, our results indicate that level of physical activity may be influenced by postoperative bariatric patients’ internalized and experienced weight-related stigma via exercise avoidance. In other words, the higher the level of weight stigma a patient had when surveyed, the more likely she or he wanted to avoid exercise, and subsequently, the less physically active she or he was. Furthermore, both experienced and internalized weight stigma only indirectly, not directly, affected PA. Studies in non-bariatric populations indicate that weight stigma may significantly lower people’s willingness to engage in physical activity [18, 20, 50]. In this sample of postoperative patients, we identified weight stigma as a factor that may be involved in patient non-compliance with physical activity recommendations, even despite the significant postsurgical weight loss our participants reported. Considering patients and medical providers’ concerns with weight recidivism after bariatric surgery, these results indicate stigma interventions may help treatment professionals identify psychosocial barriers to exercise engagement. The enduring influence of internalized weight-related stigma suggests that weight-stigma interventions might help patients meet physical activity goals at any stage after surgery, even years. This study also adds to a small but growing body of research [19, 26] demonstrating that weight stigma has important and direct negative effects on the physical health of people living with obesity, such as exercise aversion.

Our results are consistent with those of previous studies that show for people who consider themselves overweight, higher internalized stigma generates greater sensitivity to the effects of experienced stigma, making them more likely to avoid exercising and/or placing themselves in situations where experienced stigma is more likely (e.g., jogging in public, attending gyms or fitness classes) [18, 51]. In the context of our sample, higher levels of self-stigma cultivated by years spent living with high weight may generate far-reaching psychosocial effects that persist even despite significant weight loss following surgery, and these may manifest as a reluctance to engage in publically visible forms of activity [25, 52]. This is particularly important given research suggesting 200–300 min of weekly physical activity (including exercise) is necessary not only to lose weight, but to maintain weight loss over the long term [53, 54]. This is well over the often recommended 150 min of moderate-intensity physical activity to reduce health risks associated with obesity [55]. There are many known barriers to engagement in physical activity, the most common including lack of time or energy, health-related concerns (e.g., injury/fear of injury, poor health, or physical discomfort), lack of adequate social support (e.g., childcare, regular encouragement), lack of access to resources (e.g., gym, equipment, or training), and low exercise self-efficacy [56–61]. Importantly, several studies have shown a lack of motivation to be among the most prevalent reasons offered by respondents when asked why they did not engage in more physical activity [61, 62].

Our findings suggest that addressing weight stigma may help curtail this barrier. Intervention programs have successfully helped to reduce several such barriers and facilitate weight loss by targeting other psychosocial variables, such as physical activity self-efficacy or behaviors surrounding social support [63, 64]. Similar programs designed to reduce weight stigma may motivate bariatric surgery patients to be more physically active, which would help them achieve the greater overall health benefits to be gained from surgery in combination with regular exercise. As such, medical and healthcare professionals who support bariatric surgery patients may benefit from the development of pre- and post-operative programs designed to help patients cope with the psychological stresses and trauma that arose from negative interactions surrounding body size in tandem with promoting more positive views about the body and higher exercise self-efficacy [22, 64].

These results are also important for health and fitness experts who work in public spaces, particularly nonmedical facilities like gyms and fitness centers which have, in recent decades, increasingly emphasized a holistic focus on health and fitness, of which weight loss is a part [65]. To fully realize these fitness goals, it is necessary for activity centers to provide a welcoming environment that limits stigmatizing situations, ensuring that people of all body types can be motivated to exercise free from both direct and indirect forms of judgement. Habitual inactivity is considered a leading cause of non-communicable disease worldwide, with many such diseases being associated with higher weight (e.g., heart disease, high blood pressure, type two diabetes, or certain types of cancer) [66, 67]. An emphasis on cultivating stigma-free conditions in fitness centers could therefore have beneficial health outcomes for all individuals with relatively larger bodies, not just those who chose bariatric surgery as an option to manage their weight [68]. Such reduced-stigma activity centers may also lower certain barriers to physical activity and incentivize individuals to stick with long-term exercise goals even without active professional guidance.

There are several limitations to this study. First, the level of physical activity was reported in minutes as well as in days, but we could not use data reported in minutes because there were too many missing cases. Further, there remains little consensus on how to measure physical activity based on self-reported data, nor how to construct an index that reflects the combined effects of diverse activity types (e.g., vigorous and moderate physical activity and walking). Instead, a latent variable approach was used to measure physical activity based on three survey items. The World Health Organization (WHO) recommends all adults age between 18 and 65 should do at least 150 min of moderate physical activity, 75 min of vigorous physical activity, or an equivalent combination of moderate and vigorous physical activity on a weekly basis to stay healthy (more details on http://www.who.int/dietphysicalactivity/factsheet_adults/en. However, this validated index could not be used as a variable in the model due to missing cases and their negative effect on statistical power. In addition, over-reporting physical activity is a documented phenomenon especially among obese respondents, meaning the low level of reported moderate and vigorous physical activity may actually have been lower [51]. We are unsure of how systematically it was overestimated nor do we know the implication of this for the analysis. Future research could benefit from more detailed and sophisticated physical activity data collection, perhaps using passive forms of activity documentation (e.g., tracking devices, pedometers, accelerometers) that do not rely solely on self-reported estimates as these tend to be overestimated among bariatric surgery patients [35]. Second, because this project was cross-sectional in design, the findings from these results should be interpreted and discussed in terms of associations, not necessarily causations. A longitudinal study that tracks these variables as individuals move through the treatment program (e.g., pre-surgery to several years afterward) would greatly enhance our understanding of how the relationship between self-stigma, exercise avoidance, and physical activity shifts over time, particularly at the crucial 12–24 month postsurgical window when weight loss often stalls and the 2–5 year window when many patients experience weight regain [69]. This would highlight times at which certain interventions may be most beneficial. Additionally, research that focuses on developing interventions to help ameliorate the psychosocial effects of being/having been overweight would also be a logical extension of this work that may yield promising treatment outcomes for postsurgical patients specifically and weight-loss seeking individuals more generally. Third, BMI was calculated based on patients’ self-reported height and weight at the time of survey, not objectively measured height and weight collected by trained researchers. Given consistent evidence that respondents tend to under-report weight and over-report height [70, 71], this is a potential limitation of the study. However, bariatric surgery patients may be more accurate in self-reporting BMI than are other populations [70]. Last, there was a wide range of time between surgery and survey across the study participants. There might be a significant difference in observed patterns of weight stigma, exercise avoidance, and physical activity between patients who recently underwent surgery and those who did so many years ago. Subgroup analysis could not be conducted with the data due to a relatively small sample size limiting statistical power of the analysis.

## Conclusion

This study provides additional evidence that weight-related stigma has a likely effect on an individual’s capacity to lose and maintain weight via physical activity, even when they are otherwise highly motivated to do so. While more research is needed, our findings suggest that a therapeutic program designed to reduce deeply held feelings of weight-related stigma and working to create judgement-free exercise spaces may indirectly improve weight loss by decreasing an individual’s reluctance to engage in physical activity. Stigma-focused programs may therefore be a helpful complement the many nutrition- and exercised-focused programs traditionally offered to bariatric surgery patients.

## Additional files

Additional file 1: Items used to construct the WBIS Index (DOCX 13 kb)

Additional file 2: a. Structural Model, Structural Equation Model Analysis Results. b. Measurement Model, Structural Equation Model Analysis Results (DOCX 19 kb)

## Abbreviations

BMI: Body mass index
EAM: Exercise avoidance motivation
FIML: Full information maximum likelihood
MVPA: Moderate-to-vigorous physical activity
PA: Physical activity
SEM: Structural equation model
SSI: Stigmatizing situations inventory
WBIS: Weight bias internalization scale
