# Supplementary material for: Avoiding exercise mediates the effects of internalized and experienced weight stigma on physical activity in the years following bariatric surgery
Source: BMC Obes. 2018 Jul 2;5:18. doi: 10.1186/s40608-018-0195-3 (PMC6027738; doi:10.1186/s40608-018-0195-3)
Supplement: Supplementary file 1 — Items used to construct the WBIS Index (DOCX 13 kb) [file 40608_2018_195_MOESM1_ESM.docx]

| <Additional file 1> Items used to construct the WBIS Index | |
| --- | --- |
| Item | Description |
| 1 | I am less attractive than most other people if I am overweight |
| 2 | I feel anxious when I am overweight because of what people might think of me |
| 3 | Whenever I think a lot about being overweight, I feel depressed |
| 4 | I hate myself when I am overweight |
| 5 | My weight is a major way that I judge my value as a person |
| 6 | When I’m overweight, I don’t feel like my true self |
| 7 | When I am overweight, I don’t understand how anyone attractive would want to date me |
| Note: these questions were from the nineteen questions used by Durso and Latner (2008) to construct the 11-item WBIS. | |
