# Supplementary material for: Avoiding exercise mediates the effects of internalized and experienced weight stigma on physical activity in the years following bariatric surgery
Source: BMC Obes. 2018 Jul 2;5:18. doi: 10.1186/s40608-018-0195-3 (PMC6027738; doi:10.1186/s40608-018-0195-3)
Supplement: Supplementary file 2 — a. Structural Model, Structural Equation Model Analysis Results. b. Measurement Model, Structural Equation Model Analysis Results (DOCX 19 kb) [file 40608_2018_195_MOESM2_ESM.docx]

| <Additional file 2a> Structural Model, Structural Equation Model Analysis Results | | | | | | | | |
| --- | --- | --- | --- | --- | --- | --- | --- | --- |
|  | On Exercise Avoidance (EAM) | | | | On Physical Activity (PA) | | | |
|  | Est. |  | S.E. | *p*-value | Est. |  | S.E. | *p*-value |
| Exercise avoidance (EAM) |  |  |  |  | 0.159 | † | 0.087 | 0.068 |
| *Weight stigma* |  |  |  |  |  |  |  |  |
| SSI | 0.050 | ^*^ | 0.024 | 0.040 | 0.015 |  | 0.016 | 0.340 |
| WBIS | 0.411 | ^**^ | 0.135 | 0.002 | 0.037 |  | 0.126 | 0.772 |
|  |  |  |  |  |  |  |  |  |
| *Covariates* |  |  |  |  |  |  |  |  |
| Current BMI | 0.020 |  | 0.018 | 0.259 | -0.021 |  | 0.015 | 0.154 |
| Weight loss (%) | 0.011 |  | 0.010 | 0.265 | 0.012 |  | 0.009 | 0.196 |
| Time since surgery | 0.006 |  | 0.006 | 0.306 | 0.006 |  | 0.006 | 0.363 |
| Male | -0.102 |  | 0.167 | 0.540 | 0.107 |  | 0.175 | 0.542 |
| Age | 0.007 |  | 0.006 | 0.203 | 0.008 |  | 0.009 | 0.396 |
| University or above | 0.365 | ^*^ | 0.152 | 0.016 | 0.029 |  | 0.162 | 0.859 |
| High household income | -0.156 |  | 0.181 | 0.389 | -0.142 |  | 0.200 | 0.479 |
| Note: N=298;, the number of completed replications=4,856 (out of 5,000 requested); † *p* <0.10; * *p* <0.05; ** *p* <0.01; *** *p* <0.001, two-tailed; S.E. stands for standard errors. | | | | | | | | |

| < Additional file 2b> Measurement Model, Structural Equation Model Analysis Results | | | | |
| --- | --- | --- | --- | --- |
|  | Coefficient |  | S.E. | *p*-value |
| *Latent variable: exercise avoidance (EAM)* |  |  |  |  |
| Uncomfortable going to a gym | 1.000^a^ |  | - | - |
| Too many thin people at a gym | 1.128 | ^***^ | 0.172 | <.0001 |
| Embarrassed to use gym equipment | 0.919 | ^***^ | 0.168 | <.0001 |
| Embarrassed to exercise in public places | 0.910 | ^***^ | 0.142 | <.0001 |
|  |  |  |  |  |
| *Latent variable: physical activity (PA)* |  |  |  |  |
| Non-vigorous physical activity, days | 1.000^a^ |  | - | - |
| Non-moderate physical activity, days | 1.877 |  | 1.485 | 0.206 |
| No walk > 10 mins, days | 1.070 | ^***^ | 0.189 | <.0001 |
| Note: N=298; the number of completed replications=4,856 (out of 5,000 requested); ^a^ not tested for statistical significance; † *p* <0.10; * *p* <0.05; ** *p* <0.01; *** *p* <0.001, two-tailed; S.E. stands for standard errors. | | | | |
